# Supplementary material for: Are carbon emissions trading and green financial instruments synergistic? -Comprehensive quantitative research based on content analysis
Source: PLoS One. 2024 Mar 7;19(3):e0298601. doi: 10.1371/journal.pone.0298601 (PMC10919601; doi:10.1371/journal.pone.0298601)
Supplement: S1 Data — (ZIP) [file pone.0298601.s001.zip › Data/地级市Z_F工作报告.zip!/░▓╗╒/╗┤─╧╩╨/└┤╫╘╣1⁄2╓┌║┼í╛┬φ┐╦╩2╛▌═°í┐_2013.docx]

各位代表：
    现在，我代表市人民政府，向大会报告工作，请予审议，并请市政协委员和其他列席人员提出意见。
    一、2012年和过去五年工作回顾
    刚刚过去的2012年，是在严峻复杂宏观形势下克难攻坚、稳中求进的一年。一年来，全市人民在市委的坚强领导下，认真贯彻落实党的十八大及省、市第九次党代会精神，围绕科学发展、全面转型、富民强市，大力推进稳增长、调结构、转方式、惠民生、促和谐，全力保持经济社会平稳较快发展，圆满完成市十四届人大六次会议确定的目标任务。预计，实现生产总值800亿元以上，增长12%以上；财政收入163.8亿元，增长18%；固定资产投资630亿元，增长25%以上；社会消费品零售总额257亿元，增长16%；进出口总额3.3亿美元，增长38%；城镇居民人均可支配收入21000元，增长14%以上；农民人均纯收入7800元，增长14%以上。
   一年来，我们主要做了以下工作：
   （一）推进项目攻坚年，稳增长取得显著成效。重点围绕抓在建、续建项目，强化土地、征迁、融资工作，大力推进项目集中竣工和开工。全年实施项目1200个，其中竣工800个，增长24.8%。朱集矿联合试运转，7个低热值煤发电项目纳入全省规划，其中建成3个。日芯光伏一期、陕汽淮南专用车一期、北新建材等建成投产，志高动漫游园试运行，甲醇深加工等项目加快推进，淮沪二期、淮浙二期、1000千伏特高压、宝鑫新材料、锌镍蓄电池、中国移动（安徽）数据中心、国际汽车博览城等开工建设。出台加强土地房屋征收工作实施意见。全年拆迁303.8万平方米。征收土地3.9万亩。银企对接资金738亿元。全社会融资190亿元，其中五大投资公司融资74亿元、淮南矿业集团引进保险直投资金20亿元。落实国家和省促进消费、稳定外贸增长政策，加大稳增长、保企业力度，政策性减免补助31亿元。获批省困难企业349户。新增限额以上商贸流通单位124家、进出口经营权企业30家。
 （二）推进生产力布局调整，产业优化取得明显进步。启动现代农业示范园建设，推动农业向高质转变。完成现代农业示范园规划编制。出台现代农业示范园建设意见。落户园区项目30个。流转土地35.1万亩。全市“三农”支出28.2亿元，增长31.8%。全年粮食总产133.8万吨，增长5%。新增省级农业标准化示范区和示范基地6家、省级农业产业化龙头企业5家、农民专业合作社56家。认证“三品”18个。农业产业化总产值、农产品加工总值分别增长26%、30%。加快工业园区扩容升级，推动工业向高新转变。实现规模以上工业增加值450亿元，增长15%。原煤产量9100万吨，增长7.6%；发电量525亿千瓦时。市经济技术开发区完成投资32亿元，东部工业园路网建设全面启动，申报国家级开发区取得新进展。高新区完成投资21.3亿元，建成“三纵两横”路网。煤化工基地完成投资20.3亿元，综合码头等配套设施开工建设。全年建成园区标准化厂房42万平方米。企业退城进园三年计划基本完成。实施亿元以上工业技改项目48个。两化融合、云计算、物联网试点示范工作扎实推进。高新技术产业增加值、战略性新兴产业产值、非公经济增加值分别增长32%、40%、16%。加快城市新区建设，推动服务业向高端转变。出台服务业发展细则、金融业发展规划。淮南研发创意园获批省级服务业聚集区。淮矿物流园、豆腐文化产业园、新网工程等快速发展。新开工房屋面积310万平方米，竣工面积190万平方米。大八公山旅游改革试点全面启动。天宝双遗文化园建成开园。焦岗湖影视城获批4A级景区。淮南剪纸荣获全国旅游商品金奖。塌陷区综合治理取得新进展。获得世界银行贷款1亿美元。投入综合治理资金14.3亿元。搬迁安置居民2万人。
 （三）推进城乡大建设，区域统筹取得新的突破。全年完成大建设投资355亿元，增长16%。淮蚌高速公路、东西部第二通道、206国道改线、淮舜南路、朝阳东路延伸段等建成通车。合淮蚌高铁暨淮南东站建成投用。加快生态山南建设，市政务中心、武警机关新营区等7个项目竣工投用，奥体中心、中央水系公园等14个项目快速推进，连片保障房、淮师附小山南校区、观湖国际酒店等28个项目开工建设。成立滨水开发战略指挥部。加快高铁站站前广场、站前路建设。实施老城区综合改造项目10个。整治小街小巷30条。建成地下人防工程12.3万平方米。启动美好乡村建设。实施500个农村清洁工程。改造农房10480户、县乡道路70.3公里、标准化菜市场43个。加大土地整治力度，新增耕地10900亩。扎实推进新一轮治淮工程。淮河河道环境综合整治、打击非法采砂工作取得积极成效。城乡规划统筹推进，主城区20平方公里控制性详细规划、4个专项规划、4个村镇总体规划编制完成。
   （四）推进创新优势集聚，改革开放取得重大进展。加快建立“两型”体制机制，出台“两型”城市发展规划。完成城投、产投等五大投资公司市场化改革，组建市土地储备中心、市交通投资公司。招投标、国库管理、邮政监管改革不断深化。顺利启动实施“营改增”试点。顺利进入全国农汇通暨支付结算城乡一体化试点。深入推进政银合作。淮南通商农村商业银行挂牌运行。批准融资性担保机构1家，新增保险业金融机构3家。持续提升科技创新能力。完善自主创新政策。创建国家级质检中心1家、国家级企业技术中心1家、省级企业技术中心5家。煤矿安全高效开采省部共建重点实验室通过验收。建成省防爆电气优质产品生产示范区。申请专利3000件，授权专利1900件。新增省著名商标11件。全面提升对外开放水平。合肥海关现场业务处驻淮南工作组、安徽国检淮南办事处挂牌运行。成功举办第十九届中国豆腐文化节。组团参加一系列重大节会，开展海外招商，加强与央企、省企、民企、外企合作，签约项目205个。获批外贷项目资金3亿美元。全年引进内资550亿元，增长28%；利用外商直接投资1.8亿美元，增长39.8%。
 （五）推进社会事业发展，人民生活取得有效改善。全年民生投入100.8亿元，其中35项民生工程投入19.7亿元。城镇新增就业5.8万人，转移农村劳动力3.4万人。建成农民工、大学生创业园各3家。“数字物价”建设迈出新步伐。大力推进保障性安居工程建设，基本建成14834套，新开工14243套。利用住房公积金贷款支持保障性住房建设试点工作卓有成效。各项社会保险参保318万人次。新农合参合率达101.7%。城乡低保、农村五保供养标准均提高10%以上。市社会福利保障区主体工程完工。县级公立医院改革试点稳步推进。基层基本药物制度、规范药房建设取得积极成效。省市共建城乡一体化食品药品安全示范市扎实推进。大力加强文化建设。文化产业发展规划出台。淮南子传奇大型动画片在央视首播，获得国家级多项大奖。136个农家书屋、22个公共电子阅览室建成投用。少儿艺术发展项目、淮南大剧院加快推进。市文化艺术中心、工人文化宫开工建设。文物保护、非遗保护、文化精品创作、数字化电视改造取得重要进展。8人入选中国好人榜。扎实推进教育公平。学前三年入园率81%、义务教育标准化学校达标率67.7%。淮南二中新校区落成投用。校安工程全面完成。市人口和家庭公共服务中心、妇女儿童活动中心开工建设。宜家工程深入实施，人口自然增长率6.6‰。成功举办环中国自行车赛等重大赛事。淮南市志出版发行。民族宗教、外事侨务、档案工作继续加强，地震、气象、防灾、减灾水平不断提升，妇女、儿童、老龄、残疾人事业取得进步。
  （六）推进管理创新，和谐稳定开创新的局面。全国绿化模范城创建顺利通过国家核查验收。创建省级文明城市、卫生县城、卫生乡镇、生态乡村成效明显。城市管理、文明创建等志愿者服务活动蓬勃开展，数字城管不断完善，环卫作业机械化率全省领先。推进节能减排和生态环境保护。加强重点节能环保工程建设，新增污水日处理能力5万吨。单位生产总值能耗下降5%左右，化学需氧量、二氧化硫、氨氮、氮氧化物分别削减1.1%、5%、1.1%、3%。出台加强和创新社会管理实施意见。实现社区网格化管理服务全覆盖。健全完善社会稳定风险评估和应急处置机制。国家和省级示范社区建设迈出新步伐。完成第二届社居委换届选举。深入开展大走访、大接访活动，信访形势持续向好。深入推进安全大检查，整顿关闭小煤矿10对。深入做好治安整治，和谐稳定局面进一步巩固。
   各位代表！本届政府以来的五年，是全市上下奋力拼搏、奋勇前行的五年，是逆势而上、逆境崛起的五年，是战胜危机、赢得先机的五年。
——这五年，我们尽心竭力谋发展。始终坚持发展第一要务，着力推动经济持续健康发展。五年来，生产总值由350.2亿元增加到800亿元以上，年均增长12.9%。人均生产总值由15325元增加到34000元。财政收入由48.5亿元增加到163.8亿元，年均增长27.6%；财政收入占生产总值比重由13.8%提高到20.5%。规模以上工业增加值由163.2亿元增加到450亿元，年均增长18.2%。固定资产投资累计完成2045亿元，是上个五年的2.9倍，年均增长25.3%。社会消费品零售总额由109亿元增加到257亿元，年均增长18.7%。工业化率由48.6%提高到58%。主要经济指标增幅高于全国、快于全省、好于预期，经济发展的稳定性、协调性明显增强。
       ——这五年，我们潜心实干促转型。始终坚持发展必须转型、转型也是发展，加快推进结构调整和发展方式转变。全面构建“两型”城市框架体系，积极探索“四煤”发展转型路径，得到国家层面充分肯定。实施工业园区、现代农业示范园、城市新区、采煤塌陷区四大生产力布局，发展载体拓展到686平方公里。工业不断优化升级。煤电投资占比由71.1%下降到21.4%。支持淮南矿业、国投新集等大企业走出去，7户企业入围全省国税纳税50强，综合实力显著增强。原煤产量由4671万吨提高到9100万吨，电力装机容量由440万千瓦提高到948.6万千瓦，建成国家“亿吨级”煤电基地。非煤电产业在规上工业增加值中的比重由14.7%提高到19%。高新技术产业在规上工业增加值中的比重由4%提高到17%。非公经济增加值占GDP比重由33.1%提高到37%。农业产业化产值由61亿元增加到150亿元，年均增长29.2%。服务业增加值由118.9亿元增加到218亿元，年均增长10.5%。安徽现代煤化工、省级煤机装备制造等基地加快建设，创造了安徽转型发展的标志性成果。
——这五年，我们凝心聚力搞建设。始终坚持区域协调发展，促进城乡发展一体化。五年来，城乡建设投资累计完成1080.5亿元，是上个五年的10倍。公路总里程达4248公里，其中新建和改扩建公路2239公里。新增建成区面积10平方公里。新增园林绿地面积550万平方米。新区初展新姿、老区焕然一新、矿区扬眉吐气、景区创牌升级。山南山北、东部西部、河南河北统筹发展格局加速形成。高铁时代、同城效应、五彩形象日益彰显，承东启西、连南接北、内畅外联的综合优势加快构筑。以工促农、以城带乡、城乡一体走在全省前列，城镇化率由61.2%提高到65%。先后荣获中国优秀旅游城市、中国节能减排二十佳城市、中国最佳投资城市、全国地名公共服务示范市，被联合国环境规划署授予中国区环境规划示范城市优秀案例奖。
——这五年，我们真心实意抓开放。始终坚持以开放促改革、促发展，不断增强经济发展内在活力。从合淮同城迈向合淮一体，从创造性融入皖江城市带到纳入中原经济区，从国家“亿吨级”煤电基地到纳入资源型城市可持续发展规划，从“一外”为主迈向“五外”联动，战略平台日益凸显，发展机遇千载难逢。累计引进内资1420亿元，年均增长48.6%；累计利用外商直接投资5.5亿美元，年均增长29.8%。累计完成进出口总额9.5亿美元，年均增长29%。38家世界500强企业来淮设立分支机构。人才强市战略深入实施，引资、引技、引智协调互动。新一轮政府机构改革顺利完成，国有企业、事业单位、招投标改革扎实推进。文化体制改革获得全国先进。金融业资产、金融机构各项存款余额均突破千亿元。直接融资比重由22.7%上升为52.4%。创新型城市加快建设，产学研合作达到新水平。
——这五年，我们齐心协力保稳定。始终坚持安全第一、稳定为先，厚植和谐社会根基。淮河安澜、煤矿安全、社会安定的基础不断夯实。投入治淮资金16.3亿元，是上个五年的3.3倍。“五水”治理模式得到水利部肯定。淮河淮南段水质持续改善，由四类上升到三类。全社会投入安全生产资金354.5亿元，是上个五年的3.1倍。煤矿瓦斯治理淮南模式在全国推广。煤炭百万吨死亡率由2007年的0.41下降到2012年的0.13。投入社会安全资金18.1亿元，是上个五年的2.2倍。社会安全指数提升到130%。成功举办一系列重大节会，达到安全、精彩、难忘的效果。国防后备力量建设扎实有效，荣获全国双拥模范城六连冠。加强和创新社会管理，社会保持和谐稳定。
——这五年，我们诚心爱民求实效。始终坚持民生至上，多办人民满意的实事。五年来，城镇居民人均可支配收入由12003元增加到21000元，年均增长11.8%。农民人均纯收入由3697元增加到7800元，年均增长16.1%。城乡居民收入比由3.25：1缩小到2.69：1，低于全国全省比例。民生投入累计340亿元，占财政支出的76.1%，惠及全市240万群众。平均受教育年限提高到9.2年。校安工程建设，使学校正在成为最安全的地方。完善五项就业新机制，累计新增城镇就业20余万人、农村劳动力转移17万人，城镇登记失业率控制在4.5%。城镇和农村居民人均住房面积分别达31.1平方米、38平方米，分别增长21%、38.2%。医疗保险“三张网”实现全覆盖。城镇职工、被征地农民养老保险标准大幅度提高，城乡居民社会养老保险实现全覆盖。各项社会保险参保总数净增169万人。新型社会救助服务体系不断健全。
——这五年，我们用心工作抓落实。始终坚持转变政府职能，提高行政效能，不断加强政府自身建设。安徽唯一行政决策机制试点市建设扎实推进。决策、执行、监督、反馈的闭环管理机制不断完善。决策民主化进一步扩大，依法行政迈出重要步伐。提请审议地方性法规13件，制定政府规章19件。累计办理市人大代表议案、建议506件，市政协委员提案1731件，办复率100%。全面加强公务员队伍建设。政务公开实现新拓展。网络问政展现新气象。反腐倡廉取得新成效。各项工作开创新局面。
实践证明，过去五年，是我市综合实力提升最快、质量效益优化最好、城乡面貌变化最大、人民群众受益最多的时期之一。同时，我们必须看到前进道路上还有不少困难。一是发展不足、发展不优的矛盾依然存在。战略性新兴产业、现代服务业发展不足，园区经济、非公经济、县区经济仍需提速，创新能力仍需加强。二是城乡之间、区域之间发展仍不平衡。教育资源、医疗资源需要整合优化，西部、北部基础设施建设仍需加快，门户建设需要提升。三是生态治理、社会管理压力不断增大。采煤塌陷区治理任务依然艰巨，煤矿开采与环境修复矛盾仍然突出，保持煤矿安全和淮河安澜任重道远，维稳力度仍需加大。四是政府职能需要进一步转变。职能交叉不同程度存在，服务意识仍需提升，发展环境仍需改善。对此，我们将认真加以解决。
    五年的科学实践，让我们深刻认识到，保持又快又好发展，推动又好又快转型，必须坚持科学发展，打基础、管长远、增后劲。树立“功成不必在我任期”的理念，一任接着一任干，一张蓝图干到底，做到对历史负责、为现实服务、替未来着想。必须坚持改革开放，敢闯、敢试、敢为人先。强化开放第一动力、改革第一活力、科技创新第一生产力，用好两种资源、两个市场。必须坚持民生至上，问政于民、问需于民、问计于民。注重从群众中汲取智慧和力量，真正做到发展为了人民、发展依靠人民、发展成果由人民共享。必须坚持勤政廉政，公正、公道、公信。践行“五加二、白加黑”精神，甘当“一石四鸟”型干部，自我加压、自提标杆、自强不息，形成干事创业的良好氛围。
成绩来之不易，经验弥足珍贵。这是市委统揽全局、科学决策的结果，是市人大和市政协有效监督、支持配合的结果，是全市干部群众团结奋斗、辛勤劳动的结果，也是社会各界关心支持、无私奉献的结果。这里，我代表市人民政府，向全市人民，向驻淮部队、武警官兵和政法干警，向各民主党派、各人民团体和社会各界人士，向关心、支持淮南发展的同志们、朋友们，向在淮创业的境内外投资者、建设者，表示崇高的敬意和衷心的感谢！
    二、今后五年的奋斗目标和主要任务
    今后五年，是全面贯彻十八大精神、全面建成小康社会、全面转型发展的关键期。虽然国际形势复杂多变，但是我国经济长期向好的趋势没有变，安徽处于大有可为的黄金发展期没有变，淮南跨越崛起的良好势头没有变。我们要以更加宽广的国际视野、以更加科学的战略思维、以更加宏伟的气魄胆识、以更加务实的工作作风，立足新起点，抢抓新机遇，把握新要求，实现新跨越。
   今后五年政府工作的指导思想是：高举中国特色社会主义伟大旗帜，以邓小平理论、“三个代表”重要思想、科学发展观为指导，深入贯彻党的十八大精神，全面落实市第九次党代会部署，紧紧围绕主题主线，以提高经济增长质量和效益为中心，突出“两型”引领，强化创新驱动，深化改革开放，实施“四化”同步，推进“四煤”发展，加快“五城”打造，着力普惠民生，在全省率先全面建成小康社会。
    今后五年的目标是：在完成“十二五”规划任务的基础上，到2017年全面建成小康社会。转变经济发展方式取得重大进展，地区生产总值年均增长12%，城乡居民收入年均分别增长14%以上，“五城”打造迈出坚实步伐。
——建设具有全国影响力的立体能源城。统筹地下采煤、地上发电、空中利用再生能源。以建设大型煤炭基地、大型煤炭集团和大型矿井为方向，煤炭年产量达到1.3亿吨。以建设大容量、高效率和环保型的超超临界机组为方向，推进煤电联营。以低热值煤发电、生物质发电为补充，推进伴生资源循环综合利用。装机规模达到1500万千瓦。以发展再生能源、绿色能源为方向，运用高倍聚光系统发电技术，延伸光伏产业链，光伏系统及组件生产规模超过1000兆瓦，光伏装机容量达到100兆瓦。推进安全生产标准化、信息化建设。
——建设具有核心竞争力的现代产业城。统筹现代工业、现代农业、现代服务业，着力培育千亿产业、千亿园区、千亿企业。制造业规上企业关键工序数控率达85%以上。战略性新兴产业增加值占GDP比重达10%以上。围绕发展生产性和生活性服务业，着力建设大型城市综合体，加快发展大商圈、大物流、大市场。确保第三产业增加值占GDP的比重达到全面建成小康社会标准值。坚持用现代工业装备、用现代科技武装、用现代管理经营，大力推动贸工农紧密衔接、产供销融为一体。基本建成现代农业示范园。确保到2017年农业产业化总产值、农产品加工业总产值翻番。全社会科技研发经费支出占GDP的比重超过2.5%。
——建设具有一般史迹型的历史文化城。以分散在全市各处的文物古迹为主要展现方式，深入挖掘“五古三珍”资源，活化、物化、固化历史文化。全面启动历史文化名城创建工作，加快历史文化街区、文化产业园区和10个特色博物馆建设。以社会主义核心价值体系引领社会思潮、凝聚社会共识，培育核心文化和城市精神。实施文化惠民工程，打造城市10分钟文体圈和农村3公里文体圈。文化产业增加值占GDP比重达5%。
——建设具有能源城特色的优秀旅游城。按照“旅游即城市、城市即旅游”的理念，打造全国知名的旅游目的地。念好山水经，打好文化牌，做好无中生有的文章。全面提升中国优秀旅游城市的内涵和品质，完善吃住行游购娱要素配置，丰富绿色游、红色游、古色游、工业游、乡村游、休闲游、风情游等精品旅游线路，推进旅游大开发、大开放、大发展。实施“1828”工程，投资“1”千亿元以上，打造“8”大旅游区，年接待游客突破“2”千万，旅游业总收入占地区生产总值百分之“8”以上。新增5A级景区2个、4A级景区8-12个。
——建设具有永续幸福感的生态宜居城。坚持生产空间集约高效、生活空间宜居适度、生态空间山清水秀，着力推进绿色发展、循环发展、低碳发展。努力实现“五城联创”目标，提升城市文明水平。耕地面积指数达到94%以上。单位GDP消耗标准煤控制在0.84吨以下、能耗下降16%，污染减排完成省下达的目标任务。基本公共服务均等化总体实现。人民受教育程度和创新人才培养水平明显提高。人均住房使用面积达32.5平方米。城镇失业率控制在4%以内。基本社会保险覆盖率达到90%以上。环境质量指数达到100%。建成区绿地率、绿化覆盖率、人均公共绿地面积分别达40%、45%和12平方米，森林覆盖率达22%以上。社会安全指数超过100%。把淮南打造成全国煤矿城市中最洁净的城市之一。

  今后五年的主要任务是：
   （一）大力拓展“两型”发展新空间。抢抓国家“两型”社会建设机遇，争取纳入省试点范围。创建“两型”示范区。积极申报国家低碳城市试点市。推进国家首批资源型综合利用示范基地建设。加快制定产业分类核选标准和园区建设标准。完善体现生态文明要求的目标体系、考核办法、奖惩机制。在生产空间上，山南重点建高新区和低碳区，山北至淮河以南重点建加工区和服务区，淮河以北重点建重化工业区和现代农业示范区。在生活空间上，着力完善宜居宜游宜学宜业功能，深入实施保障性安居工程、千亿文化旅游振兴工程，不断提升居民文教娱乐服务支出比例，着力打造学习型城市、创业型城市。在生态空间上，着力做好加减法。在加法上，实施森林增长工程。构筑“三山鼎立”的绿色屏障体系、“三水环绕”的绿色河湖体系、“三城互动”的绿色景观体系、“三矿并举”的绿色矿区体系、“三农共建”的绿色乡村体系、“三路协调”的绿色长廊体系。在减法上，推进节能减排。加强重点节能工程建设，加大污染防治力度，坚决淘汰落后产能，提高资源节约集约水平。大力培育环保产业，积极发展循环经济，促进生产、流通、消费过程减量化、再利用、资源化。
  （二）努力构筑“四化”同步新优势。坚持立足煤、延伸煤、不唯煤、超越煤，扬煤凸企、扬名立核、扬长补短，促进工业化、信息化、城镇化、农业现代化同步发展。强化工业化主导。实施“T”型工业布局，打造沿淮经济带和合淮工业走廊。围绕低热值煤发电融入煤电基地建设，打造动力核。围绕高新区融入生态山南建设，打造信息核。围绕市经济技术开发区融入滨湖新区建设，打造支撑核。围绕煤化工基地融入北部新区建设，打造转型核。围绕合淮工业走廊淮南集中区融入合淮一体化建设，打造开放核。未来五年，完成工业投资1800亿元，新增千亿元企业1-2户、百亿元企业3-5户、亿元以上企业20户，力争规上企业达800户。强化信息化支撑。加快智慧城市顶层设计，着力建设以数字化、网络化、智能化为主要特征的智慧城市。积极争创国家云计算运用示范城市。建立“一委、一办、一中心、一智库、一园区”机制。加快物联网、云计算运用，发展电子商务、电子政务，不断提高经济社会信息化水平。强化城镇化引领。实施产城一体、旅城一体、城乡一体，做强中心城区，做大城市副中心，做优中心镇。推进城市新区开发，形成依山傍水、滨河滨湖、特色各异的城市景观。力争建成60平方公里山南新城区。积极探索行政区划调整。按照中等城市规模，打造凤台、潘集2个副中心。完善城镇规划体系，结合采煤塌陷区搬迁、行洪区退建、土地流转和农村环境连片整治，建设以工促农、以城带乡、工农互惠、充满活力的中心镇。支持民族地区、贫困地区加快发展。实施八大立体交通工程，推进“一纵一横”高速铁路、“两纵两横”高速公路、“四桥一隧”及城市外环综合交通网建设。强化农业现代化基础。启动农村综合改革示范城市建设。整合各类支农资金，统筹美好乡村、基本农田水利、现代农业示范园、农业社会化服务等建设，增强农业综合生产能力。创建稻麦“吨粮市”。实施新一轮“菜篮子”工程。大力培育农业产业化龙头企业。围绕改造提升、拆旧建新、旧村整治、特色保护，规划建设一批美食特色村、果蔬特色村、加工特色村、旅游特色村、文化特色村，推进312个美好乡村建设。
  （三）着力打造科技引领新高地。坚持科技引领发展、产业支撑发展、干部推动发展，实施创新驱动发展战略，着力推动创新资源高密聚集、创新成果高效转化、创新产业高端发展、创新文化高度自觉。坚持抓主体、抓载体、抓一体，广泛吸纳创新人才，有效激活创新资本，着力强化创新服务，建立政产学研结合的协同创新机制。坚持发展具有核心竞争力的战略性新兴产业，着力推动产业集聚和延伸，高度重视苗子型企业培育。高新技术企业达100户以上、实现产值500亿元以上。着力加强博士后工作站、院士工作站建设，大力培育国家级企业技术中心，加快建立协同创新战略联盟。到2017年，科技投入、高新技术企业、万人发明专利拥有量跻身全省第一方阵。
 （四）强力提升改革开放新水平。按照“高起点规划、高品质建设、高水平承接、高强度投入、高效率服务、高效益产出”的转型发展理念，每个省级开发区培育2—3个主导产业。充分发挥区位优势、成本优势和资源优势，全方位提升开放型经济水平。引进内资、外商直接投资、外贸进出口总额年均分别增长20%、25%、30%。围绕加快转变经济发展方式，更加尊重市场规律，更好发挥政府作用，着力破解事关发展动力的投资、消费、出口问题，着力聚焦事关转型潜力的城镇化、资本化、金融化问题。深化国有企业改革，完善各类国有资产管理体制。加快民营经济发展，非公企业突破1.5万户。培育上市企业10户。深化金融体制改革，筹建金融集团，建设金融集中区，加快发展民营金融机构。深化行政管理体制改革，创新行政管理方式，加强政府绩效管理。推进事业单位分类改革。加快财税体制改革，促进资源整合、效率提升。
 （五）奋力开创幸福和谐新生活。进一步处理好做大蛋糕与分好蛋糕、共享与多享的关系，真正把以人为本、民生优先的理念，贯穿于经济社会发展的全过程。进一步加大公共服务支出力度，更好地关注和解决就业、社会保障、城乡居民收入等问题，保障人民群众的基本需求；更好地关注和解决教育、卫生、文化等问题，满足人民群众的发展需求；更好地关注和解决安全生产、产品质量、食品药品、生态环境、社会稳定等问题，保障人民群众的安全需求；更好地关注和解决基层民主、群众自治、政务公开等问题，满足人民群众民主政治需求。多谋民生之利，多解民生之忧，在学有所教、劳有所得、病有所医、老有所养、住有所居上取得新突破。坚持教育优先、规范、均衡发展。力争学前教育入园率、中小学入学率、本科达线率分别达95%、99%、45%以上，全面实现省级义务教育基本均衡发展目标。支持职业教育、高等教育加快发展。坚持以发展促进就业，推进国家级创业型城市建设。基本完成公立医院改革。巩固完善基本药物制度。以增强公平性、适应流动性、保证可持续性为重点，全面建成覆盖城乡居民的社会保障体系。加快发展人口计生、全民健身等社会事业。推进以居家养老为基础、社区养老为依托、机构养老为支撑的养老体系建设，实现社区养老全覆盖。建立市场配置与政府保障相结合的住房制度，满足各类家庭基本需求。

 三、扎实做好2013年工作
   今年是新一届政府的开局之年。按照稳中求进的总基调，开拓创新，扎实开局。综合考虑，地区生产总值增长10%以上，财政收入增长11%以上，工业增加值增长12%以上，固定资产投资增长25%左右，社会消费品零售总额增长15%以上，城乡居民收入均增长14%左右，城镇登记失业率控制在4%以内，人口自然增长率控制在8.5‰以内，节能减排、安全生产完成年度目标任务。

  重点做好六个方面的工作：

 （一）把稳增长作为首要任务，加快培育新的增长点

   千方百计用好战略平台。成立高规格的战略平台研究机构，以政策生成项目、争取资金、扩大开放、赢得优势。发挥国家煤电基地战略平台作用，加快煤矿伴生资源综合利用，推进低热值煤电厂建设，启动局域电网布局、直供电试点。发挥皖江示范区战略平台作用，推进能源、水利、环保等大项目建设。发挥合淮一体战略平台作用，加快实施合淮工业走廊、引江济淮工程，提升淮南在“三基地、一枢纽”建设中的突出地位。发挥国家资源型城市可持续发展规划战略平台作用，加快“四煤”发展，推进采煤塌陷区综合治理和生态修复。发挥中原经济区战略平台作用，利用区域合作发展、城镇提质扩容、新农村建设、城乡土地管理等政策优势，建设“三化”协调发展先行区。

  千方百计扩大项目投资。全年完成工业投资300亿元、大建设投资400亿元。力促潘集中心选煤厂、3个输变电工程、光华光神印刷包装设备、石油化工机械、博胜新能源太阳能发电等项目开工。加快宝鑫新材料、锌镍蓄电池、陕汽淮南专用车二期、日芯光伏二期等项目建设。加强基础设施、主导产业、节能环保、社会民生等领域投资，谋划储备一批大项目。

 千方百计培育新兴消费。落实中央促进消费补贴政策，提升家电和汽车等消费能力。启动淮南东站商圈建设，延伸龙湖路地下商业街，提升西部商圈档次，加大山南商圈开发。加快十大商贸项目、十大商品市场、十大特色商业街建设，抓好城市综合体建设，推进龙湖路国际购物广场、东方国际购物中心、市经济技术开发区物流中心建设。支持大企业筹建物流中心。启动煤炭交易中心规划。加快观湖国际酒店等建设，规划建设地方名吃、旅游购物等特色街。开工建设房屋200万平方米，竣工120万平方米。启动历史文化街区和市博物馆新馆建设，推进市文化艺术中心等项目，建成淮南文化园、淮河风情文化园、2个以上特色博物馆。加大淮南子系列文化精品创作。完成有线电视数字化改造5万户。继续推进大地欢歌活动。确保文化产业产值增长50%以上。稳步推进大八公山旅游一体化，加快孙家花园原貌恢复、八公山旅游综合服务区等10个项目建设，打造晚间旅游舞台剧。启动5A级景区创建，新增4A级旅游景区1个。全年接待游客1400万人次。

 千方百计扩大融资。按照资源资产化、资产资本化、资本证券化的思路，六大投资公司完成融资100亿元以上。争取民生银行、广发证券等在淮设点。完成凤台联社改制，并实现农商行挂牌营业，推进凤台县农村金融综合改革，创建金融生态乡镇、生态村。推动2户企业上市，新增后备上市企业4户。发行中小企业集合债券、集合票据、私募债2.8亿元。设立中广淮南股权投资基金。完成小额贷款公司、融资性担保公司增资扩股工作。实现社会融资规模230亿元以上，其中新增贷款90亿元、新增直接融资50亿元。加大住房公积金支持保障房建设力度。

千方百计招商引资。突出招商主体，强化园区招商、县区招商、专业招商，确保引进内资增长20%以上、外商直接投资和外贸进出口总额均增长16%以上。突出招商平台，放大高铁效应，发挥直供电作用，拓展招商区域，推进合淮工业走廊、东西部第二通道、206国道改线、朝阳东路延伸段产业布局。突出产业链招商，围绕煤化工、煤机装备、光伏光电等战略性新兴产业，促进上下游产业集群发展。突出节会招商，高质量办好中国豆腐文化节、国际少儿艺术节，高水平参加中博会、徽商大会等节会。放大通关便利化效应，出台关于加快重点商品出口基地建设的工作意见，推进六大商品出口基地建设，增强淮化集团、中化三建、白蓝集团、振淮蓬帆等企业出口能力，培育宝鑫新材料等新的增长点。设立公共保税仓库。加强对外友好交流，以外事促进外经贸发展。

 （二）把促转型作为主攻方向，强力启动园区建设年

  加速两大基地项目集聚。壮大国家“亿吨级”煤电基地。加快沿淮经济带建设。推进杨村矿、朱集西矿、顾桥南风井、潘三矿安全改建、平电三期、淮浙二期、淮沪二期、1000千伏特高压等重大项目建设，启动潘集电厂前期工作和新集一矿技改工程。打造省级唯一煤化工基地。确保年产10万吨乙二醇工业示范装置投产，加快净水厂等配套设施建设，力促年产67万吨煤制烯烃、农民安置新区等项目开工。大力推进煤制天然气、中科合成油催化剂二期、德邦化工60万吨联碱技改等项目。

 加速工业园区转型升级。出台加快开发区转型发展的决定和园区建设年实施意见，完善开发区考核评价暂行办法。加快国家级园区、4个省级园区创建，争取4个园区纳入省级筹建范围。加快中科院淮南新能源研究中心、国产卫星应用产业园、研发创意园等项目建设，确保10个项目开工。推进市经济技术开发区扩容升级，开工建设百盟光彩大市场、亚夏汽车财富广场等13个项目，推进平安开诚、月星城市综合体、郑煤机矿用装备制造等12个项目建设，确保国瑞药业一期、凯盛重工一期等13个项目建成投产。推进合淮工业走廊错位布局、产城融合，启动合淮城际轨道交通规划建设，推动九龙大道与合肥对接，发展共建园区，对接名商名企，提升承接水平。

 加速现代农业示范园开发开放。坚持以市场的理念推进现代农业示范园建设，引进农业项目14个。投入园区发展资金10亿元，完善园区承载功能。借鉴“飞地”发展、合作共建等模式，每个园区引进2个以上大企业。推进五大农产品加工物流园建设。开工建设3个农民居住新区。完成市农科所搬迁。实现50%以上土地预流转。全市完成农业投资28亿元，储备农业项目投资规模160亿元。

 加速各类园区功能完善。完成全市园区总体规划和布局、能源基地、100平方公里煤化工基地规划。拓宽开发区发展空间，理顺开发区管委会与所在行政区的体制机制。新建标准化厂房和过渡性安置房40万平方米。确保园区工业总产值、工业增加值、税收均增长30%以上，固定资产投资增长40%以上。落实关于加快总部经济发展的若干意见。推进“千百十”工程，新增百亿元企业1户、10亿元企业2户、亿元企业6户、规模以上工业企业80户。实施技改项目200个。新增省级以上“两化”融合示范企业6户、“双软认定”企业3户、数字企业100户。

 （三）把城镇化作为主要抓手，推进城乡一体

  推动规划向农村覆盖。开展“两型”产业、园区、交通、生态等专项规划，启动淮河岸线经济发展规划、生态建设实施纲要编制。完成乡镇总体规划、172个村庄规划、城市“三旧”改造规划、城市防洪规划编制。加快编制城镇规划建设区、风景区、塌陷区范围内的村庄建设规划。出台控规编制通则、控规整合规划、主城区10平方公里控规。启动花鸟鱼虫市场规划。加强对重点区域规划管控、重要路段违法违章建筑治理。提高项目规划的验收率。

 推动城市建设向城市新区、城市副中心、中心镇拓展。推进滨高塘湖新区5平方公里路网、大通政务新区和市会展中心规划建设。加快朝阳东路、洞山东路改造，推进滨湖大道、环湖路、站前路建设，完善站前广场整体功能。启动滨淮河新区1平方公里基础设施、田家庵政务新区建设，加快广场路向北延伸。推进滨焦岗湖新区建设。启动滨瓦埠湖新区规划，完成谢家集政务新区等规划。统筹山南新区功能完善和项目推进，开工建设台湾风情园等33个项目，推进哈米王国等58个项目，建成中央水系公园、奥体中心主体育场、城市规划展示馆等项目。推进10个老城综合改造项目。启动八公山西部文化旅游产业新区建设。支持凤台、潘集实施生态县城规划建设，加速产城一体。提升11个省级中心镇、扩权强镇试点镇整体功能。

 推动美好乡村建设。坚持把现代农业作为美好乡村建设的突破口，出台扶持农业服务业发展政策措施。抓好30万亩吨粮田、60万亩小麦高产攻关示范片、70万亩水稻提升行动核心示范区、十大优质农产品生产基地建设，支持凤台建设全国水稻生产全程机械化先行县。实施畜牧业升级计划。创建省级以上蔬菜标准园2家、省级优质农产品生产基地2个。认证“三品”10个。新增市级以上龙头企业30家、农民专业合作经济组织50家。规模以上农产品加工业总产值增长10%以上。推进83个美好乡村中心村建设。完成1万户农房建设与改造任务。实施10个乡镇农村清洁工程。争取纳入省级危桥改造项目26个。加快推进大别山引水工程建设。继续实施重点洼地世行贷款项目，完成4座水库除险加固、1个中型灌区建设。启动实施架河整治等项目。推进农田水利基础设施建设。开展淮河河道采砂试点。培训新型农民1.4万人。全年脱贫2万人。

 推动立体交通建设。启动合肥新桥国际机场淮南城市候机楼建设。优化京福高铁班次，推进商杭高速铁路、济祁高速公路建设。调整改造4条省道。推进电厂路下穿、广场路下穿等7个立交项目，加快淮舜路、西部城区路网改造，建设北外环、西部第二通道，完成淮凤路、十涧湖东路等改扩建。加快孔李淮河大桥、凤台淮河二桥和6个码头、7个千吨级泊位建设。

 （四）把抓创新作为战略支撑，切实转变发展方式

 深化各项改革。建立健全政府预算体系，强化财政绩效管理，扩大参与式预算改革范围。及时跟进“营改增”试点政策。落实支持小微型企业税费减免措施。完善公务员管理制度，优化岗位设置和人员聘用。推动企业股份制改造。深化义务教育经费保障机制改革。推进中小学教师职称改革试点。依法规范招投标市场秩序。推进户籍制度改革，推行居住证制度。开展第三次经济普查。

  转化科技成果。积极争创全国科技进步先进市。推进煤制油催化剂、高浓度四氧化二氮、瓦斯治理等原始创新技术产业化，推进高倍聚光、美国微波煤炭处理及设备生产、一步法煤炭及生物质燃料制油、微生物煤制气等技术引进消化吸收再创新。加快“中国淮南煤炭交易平台”建设，启动碳汇金融研究。确保信息技术咨询服务收入增长20%，大中型企业数字化工具普及率提高3个百分点。推进煤炭开采国家工程技术研究院建设，组建省级工程技术研究中心3家。争创省级创新型试点企业2家，申报高新技术企业10家、高新技术产品20项。力争高新技术产值增长33.3%。培育毛集民营科技产业园。申请专利2040件，万人发明专利拥有量0.8件。争创全国质量强市示范市、全国防爆电气知名产品示范区，修订国家、行业标准6项，加快建设省煤电化能源计量检测中心、省煤矿安全仪器仪表检测中心。认定中国驰名商标1件、省著名商标12件。

  强化县区发展。推动“两型”县区建设。落实加快县区经济发展政策，力促各县区在全省争先进位。支持凤台工业转强、农业转精、服务业转优，对标中等城市，迈向滨河滨湖时代。支持田家庵做精一产、扩张二产、提升三产，通过高标准规划，提升幸福田家庵整体形象。支持大通放大高铁效应、滨湖效应、生态效应、园区效应，用好发源地、办好开发区、建好东大门。支持谢家集协调发展，打造宜居宜业的美好新西城。支持八公山率先实现城乡一体、旅城一体、旅游崛起、管理创新。支持潘集发挥煤电化主战场优势，推进多元发展，建设北部新区，打造副中心。支持毛集生态景区、新型城区、低碳园区“三区互动”发展。新增3个产业集群镇。

 优化民营经济。继续落实民营经济“4511”腾飞计划，出台进一步加快民营经济发展的实施细则。鼓励民间资本进入重点产业、基础设施、公用事业、民生工程等领域。实施“市场主体增量行动”，新增民营企业1500户、个体工商户9000户，非公经济增加值增长15%以上。新增省级以上中小企业公共服务平台2家。激发全民创业潮，掀起民企招商潮，推动“凤还巢”。

  简化办事流程。扎实开展依法行政示范单位创建活动。提请审议地方性法规2件，制定政府规章3件。以政务服务中心搬迁为契机，实现审批事项公开透明、办事环节封闭运行、审批权力全程监管。落实限时办理、帮办服务、一站式审批等制度。大力精简行政审批事项，市级审批项目减少一半以上，审批项目办理时限在法定时限内压缩一半以上，行政审批提前办结率达93%。

  （五）把惠民生作为根本宗旨，不断提高人民生活水平

   统筹推进民生工程。完善民生工程长效管理机制，发挥持续惠民效益。投入资金19.7亿元，实施35项民生工程。统筹煤矿和城市棚户区改造，力争新建保障性安居工程房屋3万套。解决2.43万农村人口饮水安全问题。建成50个农民体育健身工程，乡镇文化体育站实现全覆盖。稳步实施城乡居民收入倍增规划，多渠道增加居民收入。规范市场价格和收费秩序，保持物价总水平基本稳定。

  统筹治理采煤塌陷区。启动采煤塌陷区综合治理专项规划，加快城矿乡一体化发展。加大先搬后采力度，新开工项目6个，竣工4个。进一步完善补偿机制，明晰搬迁居民房屋产权，统筹住房、就业、社保、生态修复和社会管理，确保搬得出、稳得住、能致富。

  统筹解决各类群体就业。贯彻劳动者自主就业、市场调节就业、政府促进就业和鼓励创业的方针，新增城镇就业3.6万人、下岗失业人员再就业和农村劳动力转移各2万人。建成城乡创业园6个。基本实现小微企业劳动合同制度全覆盖，推行农民工工资“一卡通”。

 统筹实施素质提升行动。实施全民科学素质行动计划纲要。完成学前教育三年行动计划，新改扩建幼儿园45所。确保2个区实现省级义务教育基本均衡发展目标。完成44所标准化学校建设。大力实施名师、名校长培养工程。争创国家职业培训名城。推进安理大新校区、职教园区和体育学校综合馆建设。继续实施舜耕英才、人才青苗等工程，加大创新领军人才培养引进力度。

  统筹提高全民健康水平。建立城镇居民大病医保制度。提高新农合财政补助标准。开工建设3座病房大楼，新建扩建市中医院、市妇幼保健院，推进江淮心脑血管病中心医院建设。加强城乡卫生服务机构内涵建设。完成凤台县公立医院综合改革试点任务。办好市第七届运动会。继续加强人口计划生育工作。

  统筹完善社会保障体系。创新发展老龄事业，大力解决养老难问题。建成社区老年服务中心40个、市级公办老年公寓1所，确保社会福利保障区投入使用。出台老有所养政策，建立养老服务工作人员培训和补贴机制，启动居家养老服务平台建设。推进家政服务业市场化、产业化、社会化。抓好城乡养老保险制度衔接互认，扩大城乡居民养老保险参保率和受益率。城乡低保和五保供养标准均提高10%。筹建救灾物资储备库、灾害信息员培训中心。创建4个综合减灾示范社区。确保建制村（社区）综合服务设施覆盖率达50%。完成人防工程7.5万平方米。继续做好气象、地震、侨务、档案、民族、宗教、地方志等工作。保障妇女儿童合法权益，鼓励社会力量建设残疾人康复服务设施。

 （六）把促和谐作为重要保障，努力建设生态文明

 着力促进资源节约、环境友好。加强生态文明宣传，推进绿色低碳发展。实施50万亩农村土地综合整治，新增耕地5000亩以上。加强重点企业用能管理，实施节能与资源综合利用项目41个。加快实施城市水系改造亚行贷款项目。完善城市污水处理管网，建成潘集、毛集污水处理厂和5座村镇生活污水处理站。加大秸秆禁烧力度，提高秸秆综合利用水平。加快西部垃圾焚烧发电厂一期建设，确保燃煤火电机组综合脱硝效率达70％以上。全面开展机动车环保标志发放工作。完成5个园区规划环评。提升全国绿化模范城创建成果。成片造林2.77万亩，义务植树400万株。建成森林示范长廊42公里、绿色小康村25个。推进国家生态园林城创建。加快十涧湖湿地公园二期、焦岗湖国家湿地公园、淮西湖公园、淮河公园、舜耕山风景区建设。开展国家级生态县区、生态乡镇、生态村创建工作。启动全国文明城、全国卫生城、国家环保模范城创建。跻身省级文明城市。全面推行市容环境卫生责任区制度。开工建设餐厨垃圾收集处理项目，完成东部生活垃圾填埋场渗滤液提升工程。加大小街小巷整治力度。创建省级卫生县城、卫生乡镇各1个。

   着力强化社会和谐人人有责、和谐社会人人共享。坚持源头治理减少隐患、动态管理化解矛盾、应急处置降低风险，推进社会稳定风险评估，有效遏制各类重特大事件发生。坚持领导干部公开接访、带案下访，理旧案、清积案、防新案。坚持重心下移、警力下沉，严厉打击各类违法犯罪活动，全面加强社会治安防控体系建设，持续开展平安淮南、法治淮南创建。大力提升网格化管理服务水平，构建便捷高效、多元一体、以人为本的服务体系。加强国防动员和民兵预备役建设，增强全民国防观念，扎实开展双拥共建，促进军地融合发展，争创全省双拥模范城“八连冠”。

  各位代表！新的一年，新的希望，新的任务。我们重任在肩，务必践行为民、务实、清廉的要求，严格执行中央关于改进工作作风、密切联系群众的八项规定，凝心聚力，奋发有为。一要永葆为民的公仆底色。始终保持同人民群众的血肉联系，始终把人民利益放在第一位。围绕保持先进性和纯洁性，统筹推进创先争优、书记带头大走访等活动，深入开展调查研究，提高做好群众工作的能力。坚持多承诺，少许诺；多为民生上网，少让民众上访；多办实事，少提口号；多开门纳谏，少闭门造车；多深入基层、深入群众，少形式主义、官僚主义。二要永葆务实的时代特色。坚持实干富民、实干兴业，敢于开拓、勇于担当，努力成为攻坚的行家、克难的专家。坚持责任在先、效率优先，深化政务公开，加强效能监察，加大问责力度，进一步强化对标管理。坚持统筹兼顾、突出重点，对重点工作、重要改革、重大工程，明确目标任务，明确时间节点，明确考核内容，确保各项任务落到实处。三要永葆清廉的政治本色。牢固树立正确的世界观、权力观、事业观，坚定政治立场，明辨大是大非。自觉接受人大、政协、新闻媒体和社会各界监督，积极办理人大代表议案建议和政协委员提案，主动听取各民主党派、工商联、无党派人士和人民团体意见。抓好审计五年规划和年度计划安排，完善行政监察和行政层级监督。厉行勤俭节约，全面推进公务卡结算制度，严格公车管理、规范公务接待、加强因公出国（境）管理，确保“三公”经费零增长。全面推进惩治和预防腐败体系建设，树立干部清正、政府清廉、政治清明的良好形象。

  各位代表！站在新起点、踏上新征程、实现新跨越，我们的任务无比艰巨、我们的使命无尚光荣、我们的前景无限美好。让我们高举中国特色社会主义伟大旗帜，紧密团结在以习近平同志为核心的党中央周围，在中共淮南市委的坚强领导下，奋力拼搏，奋勇争先，奋发图强，为稳中求进、全面转型、精彩开局作出新的更大贡献！
